# Oxidative stress‐induced changes in the transcriptomic profile of extracellular vesicles

**DOI:** 10.1002/jex2.150

**Published:** 2024-04-21

**Authors:** Elizabeth R. Dellar, Claire Hill, David R. F. Carter, Luis Alberto Baena‐Lopez

**Affiliations:** ^1^ Sir William Dunn School of Pathology University of Oxford Oxford UK; ^2^ Department of Biological and Medical Sciences Oxford Brookes University Oxford UK; ^3^ Nuffield Department of Clinical Neurosciences University of Oxford Oxford UK; ^4^ Centre for Public Health Queen's University Belfast Belfast UK; ^5^ Evox Therapeutics Limited Oxford Science Park Oxford UK

**Keywords:** *Drosophila*, ExRNA, extracellular vesicles, oxidative stress, RNA

## Abstract

Extracellular vesicles (EVs) have been proposed to play dual roles in cellular homeostasis, functioning both to remove unwanted intracellular molecules, and to enable communication between cells as a means of modulating cellular responses in different physiological and pathological scenarios. EVs contain a broad range of cargoes, including multiple biotypes of RNA, which can vary depending on the cell status, and may function as signalling molecules. In this study, we carried out comparative transcriptomic analysis of *Drosophila* EVs and cells, demonstrating that the RNA profile of EVs is distinct from cells and shows dose‐dependent changes in response to oxidative stress. We identified a high abundance of snoRNAs in EVs, alongside an enrichment of intronic and untranslated regions (UTRs) of mRNAs under stress. We also observed an increase in the relative abundance of either aberrant or modified mRNAs under stress. These findings suggest that EVs may function both for the elimination of specific cellular RNAs, and for the incorporation of RNAs that may hold signalling potential.

## 1 INTRODUCTION

Extracellular vesicles (EVs) are essential in the maintenance of cellular homeostasis, with a well‐established role as a means of protein waste‐disposal (Böing et al., 2013; Emmanouilidou et al., 2010; Guo et al., 2015; Harding et al., 1984; Pan & Johnstone, 1983; Yuyama et al., 2012). From 2006, EVs were shown to contain RNA, triggering an expansion into study of the physiological role of these cellular components, both as potential intercellular messengers, through the transport of nucleic acid cargo between neighbouring or distant cells (Baj‐Krzyworzeka et al., 2006; Ratajczak et al., 2006; Skog et al., 2008; Valadi et al., 2007), and as a means of elimination of nucleic acids from cells (Chiou et al., 2018; Lasda & Parker, 2016; Squadrito et al., 2014; Takahashi et al., 2017). In recent years, the increasing accessibility of RNA sequencing technologies has revealed EVs to contain a wide range of different RNA biotypes, including messenger (mRNA), micro (miRNA), transfer (tRNA), ribosomal (rRNA), small nucleolar (snoRNA), small nuclear (snRNA) and long non‐coding (lncRNA) (Dellar et al., 2022; Lefebvre et al., 2016; Nolte‐’t Hoen et al., 2012; Tosar et al., 2015; Wei et al., 2017). Understanding the RNA content of EVs under different biological conditions is of great importance since RNA cargoes have previously been shown to influence cellular behaviour in both health and disease scenarios (Dellar et al., 2022).

Multiple studies have demonstrated that exposure to different cellular stimuli can affect EV production and function in a variety of ways (Hill et al., 2023). EV abundance has been shown repeatedly to be increased under a range of cellular stress conditions (Al‐Mayah et al., 2015; Arscott et al., 2013; Atienzar‐Aroca et al., 2016; Aubertin et al., 2016; Aubertin et al., 2016; Collett et al., 2018; King et al., 2012; Mutschelknaus et al., 2016; Salomon et al., 2013). Studies have also demonstrated changes in EV size (Bewicke‐Copley et al., 2017; Collett et al., 2018; Kore et al., 2018) and biogenesis pathways (Fan et al., 2020), as well as protein and RNA cargo (Arscott et al., 2013; Atienzar‐Aroca et al., 2016; de Jong et al., 2012; Eldh et al., 2010; Kucharzewska et al., 2013; Lee et al., 2019; Xu et al., 2015). Hence, EVs seem to play a significant role in facilitating adaptation to cellular stress conditions. However, whether this occurs through the expulsion of waste material to alleviate stress or through EV‐related signalling with surrounding cells requires further investigation, which will be addressed in future studies.

Although most EV research has been conducted in mammalian systems, EVs are also produced in bacteria, protozoa, insects and plants (Bayer‐Santos et al., 2013; Liégeois et al., 2006; Oliveira et al., 2010; Rutter & Innes, 2017; Sjöström et al., 2015). Furthermore, key aspects of EV biogenesis have been obtained through studies performed in *Drosophila melanogaster* and *Caenorhabditis elegans* (Beer & Wehman, 2017). Along these lines, Lefebvre *et al.* have recently shown that the RNA profile of EVs produced by *Drosophila* cells in culture is analogous to human cells (Lefebvre et al., 2016). In this study we made use of an in vitro system with *Drosophila* S2R+ cells to study the effects of exogenous oxidative stress on the RNA composition of EVs. Comparative transcriptomic analysis revealed the RNA content of EVs to be distinct from cellular RNA content under both normal and oxidative stress conditions. Furthermore, a subset of RNAs showed differences that are enhanced by the level of stress in a dose‐dependent manner, thus supporting an effect of stress on modifying the RNA cargo of EVs. We also observed that the relative abundance of aberrant or modified mRNAs within EVs increases upon stress, thus supporting the existence of a coupling between the RNA metabolism machinery and EV production. Together these data suggest that EVs may be used both for signalling and to eliminate specific cellular contents.

## 2 METHODS

### 2.1 Cell culture and EV isolation

S2R+ cells were maintained in Schneider's medium (Thermofisher, 21720024) supplemented with 100 IU/mL penicillin 100 μg/mL streptomycin (Fisher, 15140‐122) and 10% (v/v) heat‐inactivated foetal bovine serum (Fisher, 10500064) in T75 flasks at 25°C and ambient CO_2_. For EV extraction, cells were seeded in normal media at either 1.5 × 10^7^ or 1 × 10^7^ cells in T75 flasks. After 1 or 2 days, respectively, cells were treated with hydrogen peroxide (H_2_O_2_) (Sigma, 216763), serially diluted in media containing 10% EV‐depleted FBS and placed onto cells for 1 h, before changing to fresh media (containing 10% EV‐depleted FBS) and incubating for 48 h prior to EV extraction. EV‐depletion of FBS was carried out by ultracentrifugation in open‐topped tubes (Beckman Coulter, 344058) in a SW32Ti swing – bucket rotor (Beckman Coulter κ‐factor 204) at 120,000 g for 16 h at 4°C, then filter sterilised with 0.22 μm filter. Conditioned media was cleared of cells (10 min at 300 g), then cell debris and large vesicles (16,500 g for 20 min at 4°C) before filtering through 0.22 μm filters pre‐blocked with 0.01% BSA (Sigma, A7906) EVs were enriched using 10 mL gravity‐flow size‐exclusion columns packed with Sepharose CL‐2B (Fisher, 10217754) and PBS (containing MgCl_2_ and CaCl_2_; Sigma, D86626) buffer, voiding the first 2.5 mL and retaining 2.5 mL EVs.

### 2.2 Nanoparticle tracking analysis (NTA)

NTA was carried out using ZetaView 110 (Particle Metrix, GmbH), calibrated with polystyrene beads (Applied Microspheres) at 1:25,000. Samples were diluted to between 1:1000 and 1:250,000 in PBS such that the average number of counted particles per frame for all conditioned media was between 100 and 500. Data were acquired at room temperature with settings: sensitivity 80, shutter 100, frame rate 30, 2 cycle, minimum brightness 25, maximum size 1000, minimum size 5, tracelength 15, at all 11 positions and analysed using ZetaView software version 8.04.12.

### 2.3 Transmission electron microscopy

10 μL EV samples of ∼2 × 10^10^–3 × 10^11^ particles were incubated on glow discharged (20s at 15 mA) carbon 300 mesh copper grids (TAAB, C267) for 2 min. Samples were then stained with 20 uL 2% uranyl acetate for 10 sand visualised using a Jeol JEM‐1400 Flash transmission electron microscope with Gatan OneView 16 Megapixel camera at 100 kV.

### 2.4 Western blotting

For cellular protein extraction, the cells were washed in PBS, pelleted and snap frozen at −80°C. EV samples were concentrated to 40–200 uL in 5 kDa MWCO vivaspin 2 concentrators (Fisher 10723837). Lysis was carried out with ice‐cold RIPA buffer (Sigma 20–188) supplemented with 1:100 protease inhibitor cocktail III (Fisher P2202‐1), 1x for cell pellets and 2× for EV samples. Samples were incubated on ice for 40 min, vortexing every 10 min. Lysates were then centrifuged at 14,000 g for 15 min at 4°C. Protein content of the supernatant was quantified using Pierce BCA Protein Assay Kit (Thermofisher, 23225) at 562 nm. Western blots were run using the Invitrogen NuPAGe system. 20 μg (Latebloomer, Histone H3) or 8 μg (Actin, golgin‐84) total protein sample was prepared in LDS sample buffer (Thermofisher NP0007) (with 0.4 mM dithiothreitol for actin only) and boiled at 95°C for 5 min. Twenty microlitres samples were separated on 4%–12% polyacrylamide Bis‐Tris gels (Thermofisher, NP0322) in MES SDS buffer (Thermofisher, NP0002) for 90 min at 125 V. Protein was wet‐transferred onto nitrocellulose membrane in buffer with 15% methanol (Thermofisher, NP0006) for 2 hat 150 V at 4°C. After blocking in 5% milk in 0.1%‐PBS‐Tween for 1 h, membranes were incubated overnight with primary antibodies; late bloomer (DHSB, 10C9, 1:500) beta‐actin (Proteintech, 66009‐1, 1:5000), golgin‐84 (DSHB, 12‐1, 1:500), histone H3 (Cell Signalling, 4499, 1:1000). After washing, membranes were incubated with HRP‐labelled secondary antibodies for 1 h at room temperature. Detection was carried out with ECL Prime (Millipore Sigma, GERPN2232) on ChemiDoc MP (BioRad).

### 2.5 RNA sequencing

RNA was isolated using a Qiagen miRNeasy kit using recommended adaptations for total RNA and low expected yield, 1‐bromo‐3‐chloropropane in the place of chloroform, on‐column DNase I treatment and one additional centrifugation step (5 min at 16,000×g) to ensure complete removal of the washing buffer. Samples were eluted in 30 μL of RNAse‐free water and quantified via NanoDrop 2000c spectrophotometer for cellular and EV RNA, and Qubit RNA HS kit (Thermofisher Q32852) for EV RNA. Biological triplicates of EV and parental cell RNA were prepared and, in line with Lefebvre et al. (2016), cell samples only underwent ribo‐depletion. Dual‐indexed, strand‐specific sequencing libraries were prepared using NEBNext Ultra Directional RNA kit, and 150 bp paired end sequencing carried out on an Illumina HiSeq 4000 machine.

### 2.6 RNase protection

EV samples were concentrated to ∼60 uL in 5 kDa MWCO vivaspin concentrator (Fisher, 10723837) immediately after extraction. After storage at 4°C, samples were split into three aliquots of 15 μL. 15 μL 2% Triton X‐100 detergent (Sigma X100, diluted in PBS) or PBS was added to relevant tubes, vortexed briefly and incubated on ice for 30 min. Two microlitres RNase A was added to relevant tubes and incubated for 20 min at 37°C (final concentration 0.31 μg/uL), before adding 300 μL of Qiazol and continuing with RNA extraction as described above.

### 2.7 qPCR

Real‐time quantitative PCR was carried out using iTaq Universal SYBR Green Supermix (Bio‐Rad, 1725121). Stock solutions of each primer pair were prepared at 500 nM; then master mixes prepared for 1.1 times the necessary number of reactions for each primer set used, containing 105 μL SYBR Green, 6.5 μL nuclease‐free water and 1.55 μL primer mixes per reaction. 20 μL reactions were run in duplicate, with 18 μL master mix and 2 μL cDNA per well. Samples were placed in a CFX96 Touch thermal‐cycler (Bio‐Rad) and incubated for 30 s at 95°C, before 40 cycles of 5 s at 95°C and 30 s at 60°C, finally followed by a melt curve from 65–95°C, rising by 0.5°C at each step. For measurement of gene expression changes, the ΔΔCq method was used, with Ribosomal protein L32 used as a normaliser housekeeping gene. For RNase protection experiments, a dilution curve was carried out, and each sample expressed relative to no RNAse, no Triton treated sample.

### 2.8 Bioinformatic alignment

Quality control of sequencing data was done using FastQC Galaxy Tool (v.0.72) (Ewels et al., 2016). An rRNA FASTA sequence file was generated via Ensembl Biomart from Ensembl Genes 95; (BDGP6) (Yates et al., 2020). The following Biomart filters used were: Gene type—rRNA and Attributes—Sequences, Unspliced (Gene). BBtools (v.38.42) (Bushnell, 2014) was used to remove rRNA reads using the bbduk.sh script. Reads aligned to the *D. melanogaster* reference genome FASTA file and GTF file downloaded from Flybase (v. dmel_r6.26_FB2019 01). Reads aligned to the *D. melanogaster* reference genome FASTA file and GTF file downloaded from Flybase (v. dmel_r6.26_FB2019 01). STAR Aligner (v.2.5.3a) (Dobin et al., 2013) was used in a two‐step process; to generate the index (runMode—genomeGenerate, sjdbOverhang ‐ 149 and runThreadN ‐ 8) and align reads (out—SAMtype, outFilterMultimapNmax ‐ 1, outFilterScoreMinOverLread ‐ 0.3, outFilterMatchNminOverLread ‐ 0.3. outReadsUnmapped—Fastx, runThreadN ‐ 8). HTSeq‐count (v.0.9.1) (Anders et al., 2015) was used to generate read counts using the following parameters: position (data sorted via position using STAR as the input) and reverse (data are from a reverse strand‐specific assay). Fragment per kilobase of transcript per Million mapped reads (FPKM) values were generated using Cufflinks (v.2.2.1) (Trapnell et al., 2010) with library‐type as fr‐first strand, via command‐line in a Linux environment.

### 2.9 Bioinformatic analysis

Differential gene expression analysis on read counts was performed using DESEQ2 (Galaxy Tool v.2.11.40.2) with FDR cut‐off < 0.05 (Love et al., 2014). Gene ontology overrepresentation analysis was performed using WebGestalt in R using a custom background containing all gene identifiers detected as up or downregulated in any condition, with Benjamini‐Hochberg FDR adjustment (Liao et al., 2019). Read type distribution (Coding exons, 3′UTR, 5′UTR or intron) of aligned bam files was determined using the RSeQC tool read_distribution.py 2.6.4.1 via Galaxy server (Wang et al., 2012). The reference gene model in bed.12 format was generated from the *D. melanogaster* r6.26_FB2019_01 genome gtf file downloaded from Flybase (Larkin et al., 2021). Annotations for miRNAs and the trans‐spliced gene mdg4 were removed, then USC tools gtftoGenePred and GenePredtoBed were used to produce a bed.12 file. Percentages for read types were calculated as tags/total tags. Gene body coverage was determined using the RSeQC tool geneBody_coverage.py 2.6.4.3 with default settings: minimum mRNA length 100, using a bed.12 file containing mRNAs only (Wang et al., 2012). Quantification of coverage bias was determined using Picard tool CollectRNASeqMetrics (Broad Institute, 2009) which calculates the mean coverage of the 100 most 3′ or 5′ bases divided by the mean coverage of the whole transcript. Base mismatch rate was determined using the Picard AlignmentSummaryMetrics tool on bam alignment files (Broad Institute, 2009).

### 2.10 Statistics

Statistical analysis was carried out via Graphpad Prism (v9.4.1) unless otherwise stated. Volcano plot was generated from DESEQ2 data using the bioinfokit Python package (v2.0.8) (Bedre, 2022).

### 2.11 Data availability

The data for this study have been deposited in the European Nucleotide Archive (ENA) at EMBL‐EBI under accession number PRJEB65886 (https://www.ebi.ac.uk/ena/browser/view/ PRJEB65886).

## 3 RESULTS

### 3.1 Hydrogen peroxide stress increases production of EV‐like particles

**FIGURE 1:**
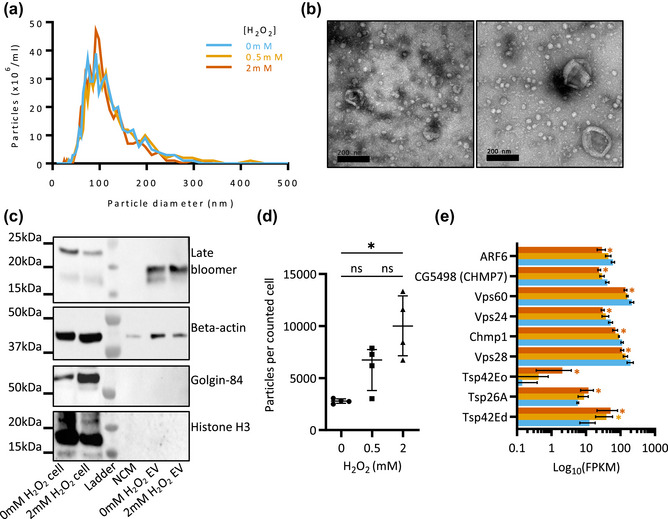
Increased EV‐like particles produced under hydrogen peroxide stress. (a) NTA particle counts of EVs from S2R+ cells subjected to 0,0.5 or 2 mM H_2_O_2_ stress. One biological replicate shown. (b) Representative TEM of negatively stained EVs under 0 or 2 mM H_2_O_2_ stress. (c) Representative Western blots for EVs and cells under control and 2 mM H_2_O_2_ stress. Non‐conditioned media (NCM) contained 10% depleted FBS. (d) NTA particle count normalised to cell count at the point of conditioned media collection. Graph shows median and interquartile range of four biological replicates, significance was assessed with Kruskal–Wallis test followed by Dunn's multiple comparisons test (**p < 0.01). (e) FPKM abundance values in cells for tetraspanin and ESCRT complex mRNAs that were significantly altered in 2 mM H2O2‐treated cells relative to non‐treated cells. Graph shows mean and standard deviation for three biological replicates, with differential expression determined by DESEQ2 (FDR < 0.05).

In order to study the effect of oxidative stress on EV biogenesis and RNA cargo, we made use of the adherent *Drosophila* S2R+ cell line (Lefebvre et al., 2016; Parchure et al., 2015), cultured in media containing EV‐depleted FBS, and stressed with hydrogen peroxide (H_2_O_2_), at 0, 0.5 or 2 mM concentrations (Figure S1) (Atienzar‐Aroca et al., 2016; Radyuk et al., 2006). Whilst FBS is known to contain bovine‐derived EVs and lipoproteins (even after EV‐depletion), we found that alternative serum‐free culture or defined supplements resulted in the S2R+ cells appearing highly stressed and apoptotic, so could not be used as a control condition (Figure S2). Profiling of small EVs showed peak particle size from 70 to 120 nm under all three conditions by nanoparticle tracking analysis (NTA), with typical EV morphology by transmission electron microscopy (Figure 1a, b). Western blotting confirmed the presence of common EV markers such as the tetraspanin (Latebloomer) and β‐actin (González‐Méndez et al., 2020; Hoshino et al., 2020; Koles et al., 2012; Linnemannstöns et al., 2022) (Figure 1c, Parchure et al., 2015). Importantly, the absence of golgin‐84 and histone‐H3 indicated no contamination from other cellular components under both control and high stress conditions. We then evaluated whether the number of EVs was altered upon stress, observing a significant increase in particle number per cell (Figure 1d). Furthermore, transcriptomic analysis of the parental cell RNA showed significantly altered expression of multiple known components of EV biogenesis pathways (Figure 1e). Notably Tsp42Ed, the closest human ortholog of which is CD63, was significantly upregulated in both 0.5 and 2 mM H_2_O_2_ stress conditions, further supporting the case for increased EV biogenesis under stress.

### 3.2 Differential mRNA distribution in EVs and parental cells under stress

**FIGURE 2:**
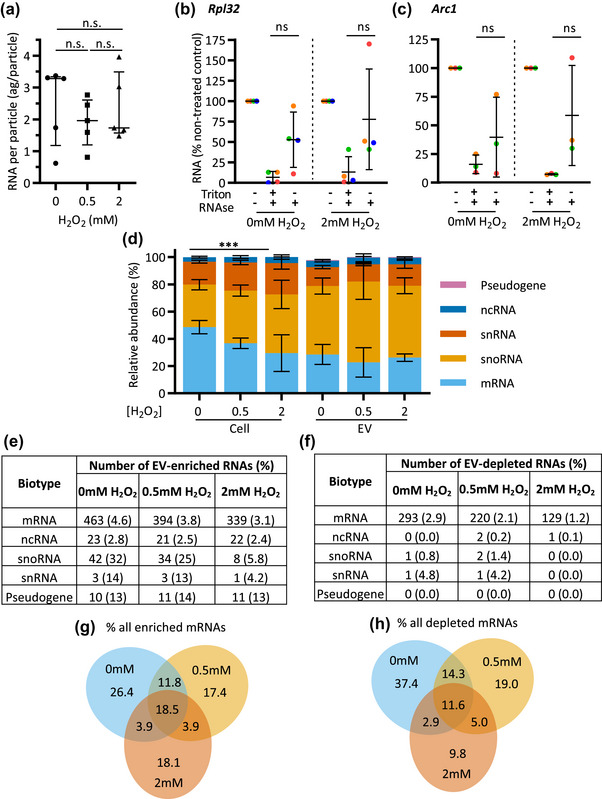
**EV RNA is distinct from cellular RNA and is altered under oxidative stress**. (a) RNA yield (attograms per particle) quantified by Qubit HS RNA after normalisation to NTA particle count. Graph shows median and interquartile range of five biological replicates, significance was assessed with Kruskal–Wallis test followed by Dunn's multiple comparisons test. (b, c) RNAse protection assay showing qPCR results for *Rpl32* and *Arc1* after treatment with RNAse A alone, or when pre‐treated with Triton X‐100 detergent, for EV samples derived from non‐treated or 2 mM H_2_O_2_‐treated cells. cDNA synthesis carried on 15–40 ng of RNA from untreated control or equivalent volumes for RNAse A/Triton X‐100 treatments. Graphs show mean and standard deviation for four or three biological replicates, respectively. Statistical significance assessed with two‐way ANOVA followed by Sidak's multiple comparisons test. (d) Relative proportions of each RNA biotype in each sample, showing percentage of total FPKM mapping to each biotype in the top 1000 most abundant RNAs. Graph shows mean and standard deviation of three biological replicates, and significance assessed with two‐way ANOVA followed by Dunnet's multiple comparisons test. (e, f) Percentage of RNAs showing enrichment or depletion in EVs derived from non‐treated, 0.5 mM H_2_O_2_ and 2 mM H_2_O_2_‐stressed cells relative to cells, stratified by biotype. (g, h) Venn diagrams showing overlap of significantly enriched or depleted mRNAs, expressed as a percentage of total enriched RNAs under any condition.

We next carried out an analysis of the RNA associated with the EVs in our experimental paradigm. Quantification of total RNA (by Qubit fluorometric assay) indicated that the amount of RNA, normalised to the total particle number, remained unaffected by the level of stress (Figure 2a). This RNA then underwent sequencing without any prior selection or depletion for procedures, alongside ribo‐depleted RNA from matched cell samples. Reads mapping to ribosomal, transposon and *Drosophila* virus‐derived RNAs were highly abundant in EV‐associated RNA as seen in data from previous studies (Ashley et al., 2018; Lefebvre et al., 2016), but did not show notable differences in response to stress (Figure S3). Using qPCR of RNAse‐treated RNA in the presence or absence of detergent, we confirmed that a portion of two highly abundant mRNAs *(Activity‐regulated cytoskeleton associated protein 1* ‐ *Arc1* and *Ribosomal protein L32* ‐ *Rpl32*), was protected from degradation by the lipid membrane, indicating it to be EV‐internal RNA cargo, although not eliciting whether these mRNAs were present in a full‐length or fragmented state (Figure 2b, c). Furthermore, despite significant variability among samples, the proportion of RNAse‐sensitive RNA remained comparable between the control and stress conditions (Figure 2b, c).

To characterise the nature of RNAs associated with EVs, we carried out biotype analysis of the remaining *Drosophila*‐genome‐mapped RNA for the top 1000 most abundant RNAs captured in both cell and EV samples. In cells mRNA was the most abundant biotype under control conditions (49 ± 4.9%) and was significantly reduced by 39% under 2 mM H_2_O_2_ stress (*p* < 0.001) (Figure 2d); however, this difference was not observed in EV samples (Figure 2d). The relative abundance of snoRNAs in all EV samples (54 ± 8.7%) was also markedly high compared to cells (37 ± 7.8%) (Figure 2d). These data indicate that the presence of RNA associated with EVs is not solely a result of cellular RNA abundance, and instead suggest a mechanism of specificity that is modified under stress. We then carried out differential distribution analysis of EV and cell samples under each condition to identify which specific transcripts were localised to EVs. In total 837 (control), 691 (0.5 mM H_2_O_2_) and 514 (2 mM H_2_O_2_) RNAs were unevenly distributed, which were predominantly mRNAs (756 of the total in control conditions; Figure 2e, f). However, significant EV‐enrichment of specific RNAs could be detected for all biotypes, with snoRNA and snRNA enrichment detected at a high rate under control conditions (Figure 2e).

As mRNAs make up the largest biotype group, we then looked to compare the overlap between the differential distribution of specific mRNA transcripts between conditions. Irrespective of the stress levels, 18.5% (141 mRNAs) of total mRNAs were always significantly enriched in EVs (Figure 2g). 18.1% (138) were specifically enhanced in EVs under high levels of stress (2 mM H_2_O_2_ alone), and only 3.9% (30 mRNAs) in both low and high stress conditions (0.5 and 2 mM H_2_O_2_) (Figure 2g). A similar trend was observed for the mRNAs specifically depleted in EVs (Figure 2h). To investigate the molecular nature of stress‐specific EV enriched and depleted mRNAs, we performed a bioinformatic GO term analysis. Amongst mRNAs enriched in EVs under any condition, terms relating to development and morphogenesis were highly overrepresented. This overrepresentation was more pronounced for the mRNAs that were EV‐enriched only under the higher level of stress (Supplementary Data). For mRNAs that were EV‐depleted specifically under high stress, the most overrepresented terms related to regulation of MAPK signalling (Supplementary Data). The mRNAs in this MAPK signalling category did not show significant differences in expression in cells under stress, so their depletion in EVs may reflect increased cellular retention that is important in stress response (Figure S4).

### 3.3 Stress EVs show enrichment of stress‐related mRNAs compared to control EVs

**FIGURE 3:**
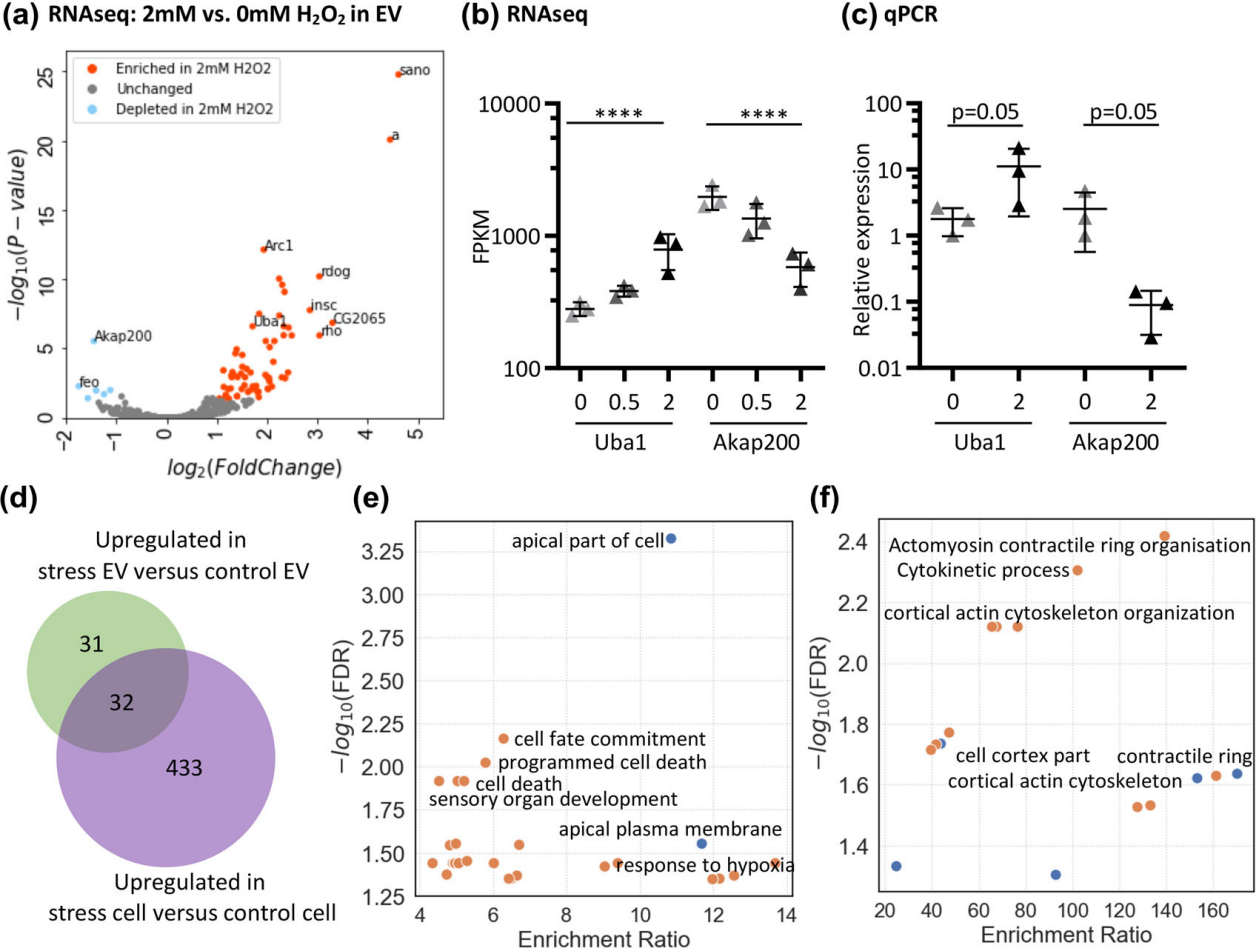
A subset of EV mRNAs show stress‐dose responsive changes in abundance. (a) Volcano plot showing differential abundance results of all RNA in 2 mM H_2_O_2_ EVs compared to 0 mM EVs from DESEQ2 analysis. (b) RNA sequencing data and (c) qPCR data for Uba1 and Akap200, normalised to Rpl32 housekeeping gene. Mean and standard deviation for three biological replicates, statistical significance assessed by one‐tailed Mann Whitney test. (d) Venn diagram showing overlap of mRNA significantly upregulated in 2 mM H_2_O_2_ EVs versus non‐treated EVs, and mRNAs significantly upregulated in 2 mM H_2_O_2_ cells versus non‐treated cells. (e, f) Gene ontology analysis of mRNAs enriched or depleted in 2 mM H_2_O_2_ derived EVs compared to non‐treated EVs.

Given the evidence that mRNAs can be specifically localised to EV or cell under stress, we wanted to explore the potential functional significance of such changes. To do so we compared the mRNA content between EV samples, rather than EV with cell. Sixty three mRNAs were significantly upregulated and seven significantly downregulated in EVs generated under 2 mM H_2_O_2_ stress compared to control EVs (FDR < 0.05) (Figure 3a). qPCR analysis of two EV mRNAs, *Ubiquitin activating enzyme 1 (Uba1)* (significantly enriched 3.3‐fold) and *A kinase anchor protein 200* (*Akap200)* (2.7‐fold depleted) showed differential expression between EVs under stress, confirming the transcriptomic data (Figure 3b, c). Under 0.5 mM H_2_O_2_ stress, only one mRNA (*Arc1)* reached the threshold of significance, but comparison of abundance values demonstrated a trend for upregulation of the 63 mRNAs upregulated under high stress, indicating that these changes are stress‐dose‐dependent, with biological relevance (Figure S5). Furthermore, 49% (31/63) of up‐regulated RNAs and all seven downregulated RNAs, were exclusive to EVs, as were not significantly altered in stress cells compared to controls (Figure 3d). We next explored the functional nature of these enriched targets. mRNAs linked to cell fate commitment, response to hypoxia, and programmed cell death were amongst the categories overrepresented within the 63 upregulated mRNAs in EVs, whilst the seven downregulated mRNAs related exclusively to cytoskeletal functions (Figure 3e, f). Taken together, these results demonstrate that stress‐cell derived EVs contain mRNA cargo which has functional importance, but does not confirm whether these are linked to full‐length or fragmented mRNAs.

### 3.4 Enrichment of fragmented mRNA in EVs under oxidative stress

**FIGURE 4:**
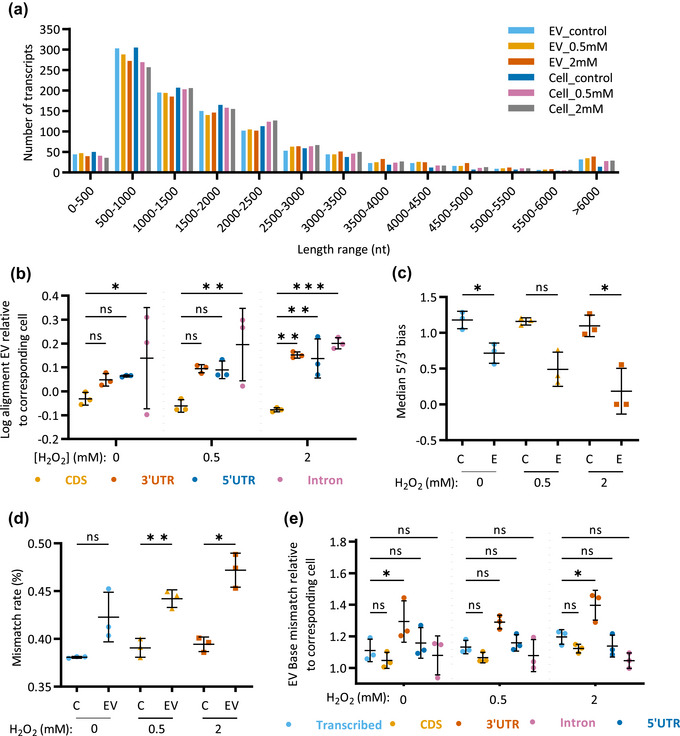
Enrichment of introns, UTRs and base‐mismatched mRNA in EVs under oxidative stress. (a) Length distribution of the top 1000 most abundant mRNAs (excluding extremely highly abundant *Arc1*) detected in EV and cell samples. (b) Enrichment of each gene region in EVs derived from non‐treated, 0.5 mM H_2_O_2_ and 2 mM H_2_O_2_‐stressed cells against parental cell samples. (c) 5′/3′ bias of all samples, representing the ratio of mean coverage of the 100 most 5′ bases divided by the mean coverage of the whole transcript, to the mean coverage of the 100 most 3′ bases divided by the mean coverage of the whole transcript, for the 1000 most abundant transcripts. (d, e) Mismatch rate for sequencing read alignments in (d) all regions of each sample, or (e) in each gene region in EVs relative to parental cell samples. C: Cell. Graphs show mean and standard deviation for three biological replicates, significance assessed with one‐way or two‐way ANOVA followed by Dunnet's multiple comparisons test.

Beyond the functional enrichment, we also investigated whether there were biophysical factors facilitating the incorporation of specific RNAs in EVs. Since several studies have proposed a size limitation on EV‐RNA (de Jong et al., 2020; Hinger et al., 2018; Mosbach et al., 2021; Shurtleff et al., 2017; Wei et al., 2017), we analysed the length of the mRNAs present in our sequencing data. Due to low read depth in the EV samples, we considered the median length of all known splice variants for each identified gene rather than individual variants, for the top 1000 most abundant mRNAs. Irrespective of the stress levels, the length distribution of mRNAs detected in cells and EVs was comparable, with no preference for shorter transcripts in EVs (Figure 4a). To evaluate whether such transcripts were intact or just corresponded to fragments of the full‐length mRNA, we evaluated the read distribution across the gene body. Our data unveiled that under stress there was a significant enrichment of introns, 3′UTR and 5′UTR regions relative to the coding region (CDS), examples of which were confirmed using Integrative Genome Viewer (IGV) (Figure 4b, Figure S6). Quantitative assessment of total gene coverage also showed a significant 5′/3′ end bias in EV‐associated RNA that was greatest under stress (Figure 4c). Together these results suggest that the specific mRNA fragments are incorporated into EVs.

### 3.5 Increased mismatch rate in 3′UTRs in EVs under oxidative stress

Given the tendency for low alignment rates in EV samples reported by others (Cha et al., 2015; Hinger et al., 2018; O'Grady et al., 2022; Tosar et al., 2015; Yang et al., 2017), the quality of sequencing read alignment was further explored. The base mismatch rate of each sample relative to the reference sequence was higher in EV‐associated RNA compared to cell RNA and this trend was further enhanced under high stress conditions (Figure 4d). Region‐specific evaluation revealed that these mismatches were relatively enriched in 3′UTRs compared to the total transcribed region of mRNAs (Figure 4e). Although differences in read depth between cell and EV samples prevented us from comparing the rates of specific sites of mismatches, our data have revealed a complex interplay between EV‐associated RNA, RNA decay and RNA modification.

## 4 DISCUSSION

EVs have been described as having roles in both intercellular communication and in cellular waste disposal, but the importance of these functions in different cell types and different cellular contexts is under debate (Dixson et al., 2023). Here we show that there is specific enrichment of some RNAs in EVs, and that this changes in a dose‐dependent manner in response to oxidative stress. These changes indicate that the production of EVs is of enhanced biological relevance under conditions of cellular stress.

Our data shows an increase in EV production in *Drosophila* S2R+ cells in response to hydrogen peroxide treatment. Similar results have been seen in studies of some mammalian cell types in response to a variety of oxidative‐stress inducing conditions (Atienzar‐Aroca et al., 2016; Hedlund et al., 2011; Wang et al., 2021), and more broadly under cellular stress (Al‐Mayah et al., 2012; Arscott et al., 2013; Aubertin et al., 2016; Collett et al., 2018; King et al., 2012; Mutschelknaus et al., 2016; Salomon et al., 2013). In transcriptomic analysis of cell RNA under oxidative stress we observed changes in expression of genes involved in regulating EV production, including several tetraspanin proteins (Tsp42Ed, Tsp42Eo, Tsp26A), and ESCRT‐III components (CHMP1, CHMP7, VSP24, VSP60). Tsp42Ed was recently identified as highly enriched in proteomic analysis of *Drosophila* haemolymph EVs, whilst expression of the closest mammalian homolog, CD63, has been demonstrated to influence rates of EV production (Hurwitz et al., 2016; Linnemannstöns et al., 2022; Sung et al., 2020). Tsp26A and Tsp42Eo expression were also upregulated in response to manganese chloride‐induced stress, whilst ESCRT‐III proteins have been specifically implicated in regulating EV biogenesis under cellular stress (Marie et al., 2023; Mohr et al., 2018). These data therefore suggest EV release to be an important aspect of cellular stress response, whether by providing a means for a cell to expel unwanted material under stress, or as a mechanism for interaction with surrounding cells.

Current evidence suggests little difference in total RNA content between bulk EV subpopulations but distinct differences in their composition (Barman et al., 2022; Crescitelli et al., 2013; Hardy et al., 2019; Lässer et al., 2017). Here we profiled the RNA cargo of small EVs from *Drosophila* cells and found no significant difference in the bulk RNA content under oxidative stress, but changes to the biotype composition. In contrast to the low abundance in mammalian EVs (Baglio et al., 2015; Chiou et al., 2018; Lefebvre et al., 2016; Nolte‐’t Hoen et al., 2012; Tosar et al., 2015; Wei et al., 2017), snoRNAs appear to be highly abundant in *Drosophila* EVs (Lefebvre et al., 2016) and our own data (Lefebvre et al., 2016). However, with lengths ranging from 46 to 316 nt length in *Drosophila*, snoRNAs are likely incompletely represented within our 150 bp paired‐end sequencing data, so the difference observed may be unique to specific snoRNA subsets. Given the importance of cellular snoRNA localisation in regulating stress response (Holley et al., 2015; Lee et al., 2016; Stepanov et al., 2015), and recent identifications of differentially abundant EV‐snoRNAs in pathological states (Anderson et al., 2022; James et al., 2021), *Drosophila* cells could provide an ideal model system to investigate the biological significance of EV‐incorporated snoRNAs in the future.

It is now well‐established that it is possible for mRNA within EVs to be delivered to recipient cells and subsequently translated (Albanese et al., 2021; Hinger et al., 2018; Lai et al., 2015; Ratajczak et al., 2006; Ridder et al., 2015; Ridder et al., 2014; Skog et al., 2008; Usman et al., 2018; Valadi et al., 2007; Zomer et al., 2015). However, there is a need to understand which mRNAs are transported, how frequently this process is occurring in vivo, and whether this frequency is modified under different cellular conditions (Dellar et al., 2022). We were able to detect mRNAs of a range of lengths by qPCR (*Rpl32* ‐ 524–747 nt, *Arc1 ‐* 2371 nt and *Uba1* ‐ 4343 nt) and saw no preference for reads from short mRNAs in transcriptomic data, in both resting and oxidative stress conditions, indicating that EV RNA signalling is not limited to small RNAs as has sometimes been proposed (de Jong et al., 2020; Hinger et al., 2018; Mosbach et al., 2021; Shurtleff et al., 2017; Wei et al., 2017). Our data indicated a strong enrichment of mRNAs relating to developmental functions in all EVs, especially under conditions of stress. Additionally, stress‐response functions were overrepresented within the subset of mRNAs enriched in stress EVs compared to control EVs. This finding is consistent with the hypothesis that EV mRNA cargo possesses significant signalling potential *if* taken up into, and translated in, surrounding cells. Although a direct comparison to previous studies was not possible due to experimental differences, we highlight that enrichment of mRNAs involved in stress response mechanisms has previously been reported (de Jong et al., 2012). However, we do note that in our study, and the cited studies, the changes detected likely occur at the latest stages of the homeostatic response rather than early, transient stress response. Nevertheless, given that stress conditions have also been shown to increase uptake and/or delivery of mRNAs in vitro and in vivo (Haimovich et al., 2017; Mutschelknaus et al., 2016; Ridder et al., 2014; Valkov et al., 2021), any full‐length transcripts from this later stage response may have particular significance in the vicinity of stressed cells.

Our data have not, however, confirmed the presence of full‐length transcripts in EVs. On the contrary, several lines of evidence indicated that a fraction of mRNAs present in EVs were fragmented, aberrant, or modified, and that this fraction was higher upon stress treatment. In line with previous literature, EV‐enriched RNA reads frequently mapped to introns and UTRs (Hardy et al., 2019; Li et al., 2013–Kossinova et al., 2017; Nolte‐’t Hoen et al., 2012; Wei et al., 2017). Whilst such fragmented mRNAs would not result in the translation of canonical protein, this does not preclude them from holding signalling roles in recipient cells. Multiple studies have reported the regulated production of isolated UTR fragments in human, mouse and *Drosophila*, which may be translated to produce small peptides (Kocabas et al., 2015; Mercer et al., 2011; Starck et al., 2016; Sudmant et al., 2018). The RNAs produced may also act in a regulatory manner, as “molecular sponges” for other *trans‐*acting RNA binding proteins or microRNAs (Mercer et al., 2011). The subset of mRNAs reported as showing high production of isolated 3′UTRs show functional enrichment for developmental gene ontology terms, whilst an enrichment of isolated 3′UTRs was also seen under oxidative stress (Kocabas et al., 2015; Sudmant et al., 2018). Given the enrichment of UTR regions that we observe in EVs under oxidative stress, and the enrichment for developmental and stress functions of mRNA, it is conceivable that these fragments may hold intercellular signalling functions.

Alongside enrichment of intronic and UTR regions, we also observed a higher base mismatch rate in EVs under stress. This observation may be due to the presence of non‐templated RNA base modifications that alter the length and sequence of the cDNA products of reverse transcriptase (Hinger et al., 2018). RNA is highly vulnerable to oxidative damage, particularly the oxidation of guanine to 8‐oxoguanine (8‐oxo‐G), so an increase in defective RNA may explain the higher rate of mismatch we observe in stress EVs. The sensitivity of different RNA biotypes to oxidative damage has not been well studied, so this may also underly the apparent difference in biotype abundance that we have observed. However, 8‐oxo‐G is estimated to occur at a rate of 0.001%–0.01%, even under oxidative stress, whilst other modifications that hold functions in mRNA regulation are more widespread, with the most common N6‐Methyladenosine (m6A), occurring at an estimated rate of 0.15%–0.6% (He & He, 2021; Hofer et al., 2005). m6A has higher prevalence in mRNA under stress and has a role in stress response through the localisation of mRNAs to stress granules (Anders et al., 2018; Fry et al., 2017). Given that a higher prevalence of m6A‐modified RNA has been observed in EVs (and other extracellular RNA) in *Arabidopsis*, an increase in the rate of m6A may also explain the increased rate of base‐mismatch observed in our stress EVs (Zand Karimi et al., 2022). Further study to profile EV RNA by long‐read and modification‐sensitive sequencing techniques will be necessary to elucidate which base‐modifications are present, and whether each biotype of RNA is present in an intact or fragmented state, thus uncovering its’ likely biological significance. In either case, however, our data indicate that these changes are most evident under conditions of cellular stress.

In conclusion, our study demonstrates that there are changes in EV RNA profiles in response to oxidative stress that are compatible with the existence of specific RNA waste disposal and signalling functions for EVs. However, the relative biological significance of these contrasting roles is likely to be highly context dependent. It is also important to note that even “waste” and degraded RNA may hold strong signalling potential; acting as damage‐associated molecular patterns to activate toll‐like receptors (Fabbri et al., 2012; Moroishi et al., 2016; Nabet et al., 2017). Future work, out of the scope of this manuscript, is needed to fully determine the biological significance of aberrant and/or modified RNAs incorporated into EVs under stress conditions in different biological scenarios.

## AUTHOR CONTRIBUTIONS

**Elizabeth R. Dellar**: Conceptualization (equal); data curation (equal); formal analysis (equal); investigation (equal); methodology (equal); project administration (equal); visualization (equal); writing—original draft (lead); writing—review and editing (lead). **Claire Hill**: Conceptualization (equal); data curation (equal); formal analysis (equal); investigation (equal); methodology (equal); validation (equal); visualization (equal); writing—review and editing (equal). **David R. F. Carter**: Conceptualization (equal); funding acquisition (equal); investigation (equal); methodology (equal); supervision (equal); writing—review and editing (equal). **Luis Alberto Baena‐Lopez**: Conceptualization (equal); funding acquisition (equal); investigation (equal); methodology (equal); supervision (equal); writing—review and editing (equal).

## CONFLICT OF INTEREST STATEMENT

D.R.F.C. is an employee of Evox Therapeutics. The other authors declare no competing interests.

## Supporting information

Supporting Information

Supporting Information
