# Supplementary material for: Oxidative stress‐induced changes in the transcriptomic profile of extracellular vesicles
Source: J Extracell Biol. 2024 Apr 21;3(4):e150. doi: 10.1002/jex2.150 (PMC11080704; doi:10.1002/jex2.150)
Supplement: Supplementary file 1 — Supporting Information [file JEX2-3-e150-s001.pdf]

Oxidative stress-induced changes in the transcriptomic profile of extracellular vesicles

Elizabeth R Dellar [#1,2,3], Claire Hill [1,4], David R.F Carter[2, 5], Luis Alberto Baena-Lopez[#1]

Affiliations:

[1] Sir William Dunn School of Pathology, University of Oxford, South Parks Road, Oxford, UK.

[2] Department of Biological and Medical Sciences, Oxford Brookes University, Gipsy Lane, Oxford, UK.

[3] Nuffield Department of Clinical Neurosciences, University of Oxford, Oxford, UK.

[4] Centre for Public Health, Queen's University Belfast, Belfast, UK.

[5] Evox Therapeutics Limited, Oxford Science Park, Oxford, UK.

[#] Corresponding authors: elizabeth.dellar@ndcn.ox.ac.uk and alberto.baenalopez@path.ox.ac.uk

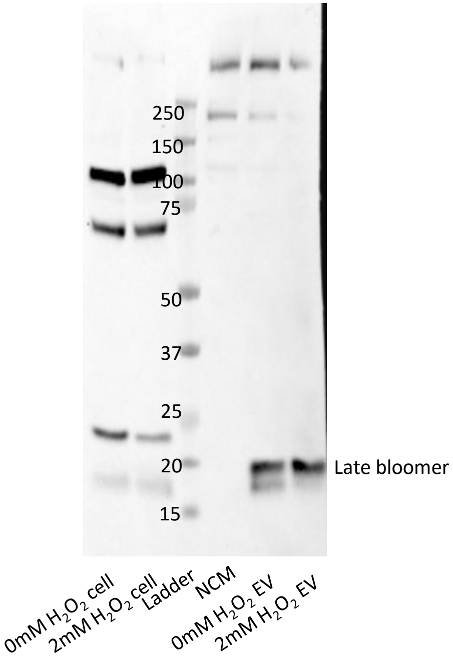

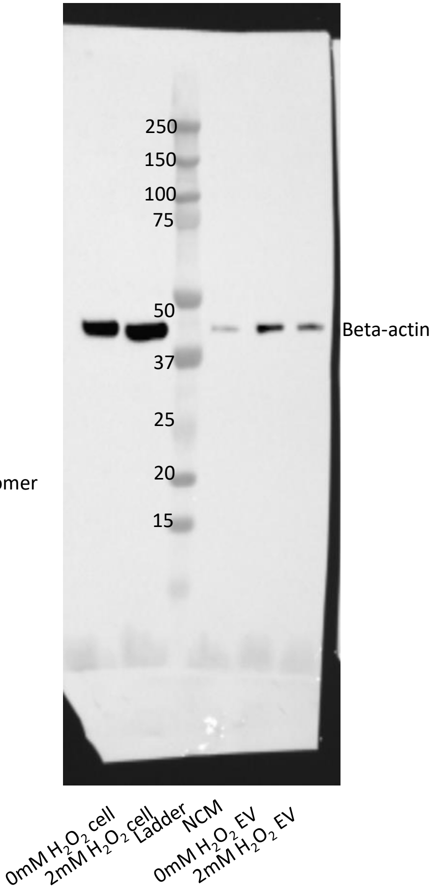

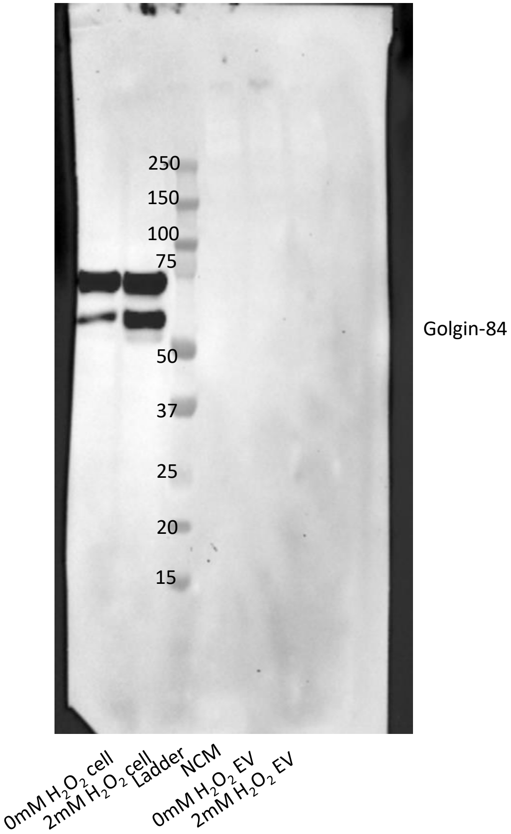

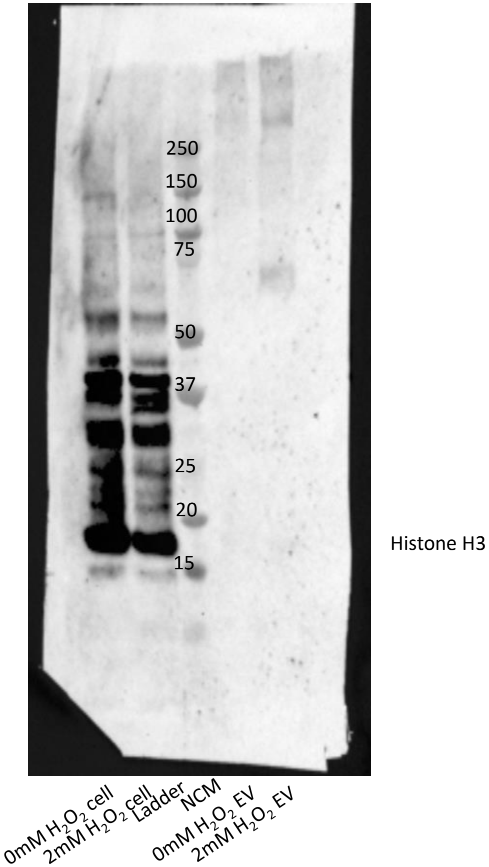

Full-size blots from Figure 1

(A)

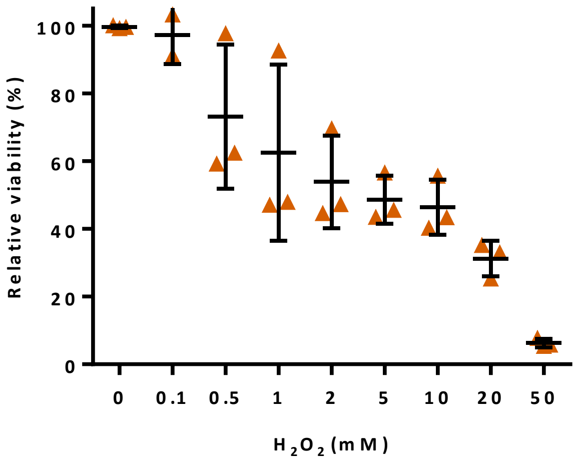

Supplementary Figure S1: Viability curve for H_2O_2 treatment

Viability curve for S2R+ cells using resazurin assay, after one-hour treatment with H_2O_2 diluted in media with 10% depleted FBS and 48-hour incubation. Median and interquartile range of three biological replicates shown. Each point the mean value of technical replicates, normalised to control condition for that biological replicate.

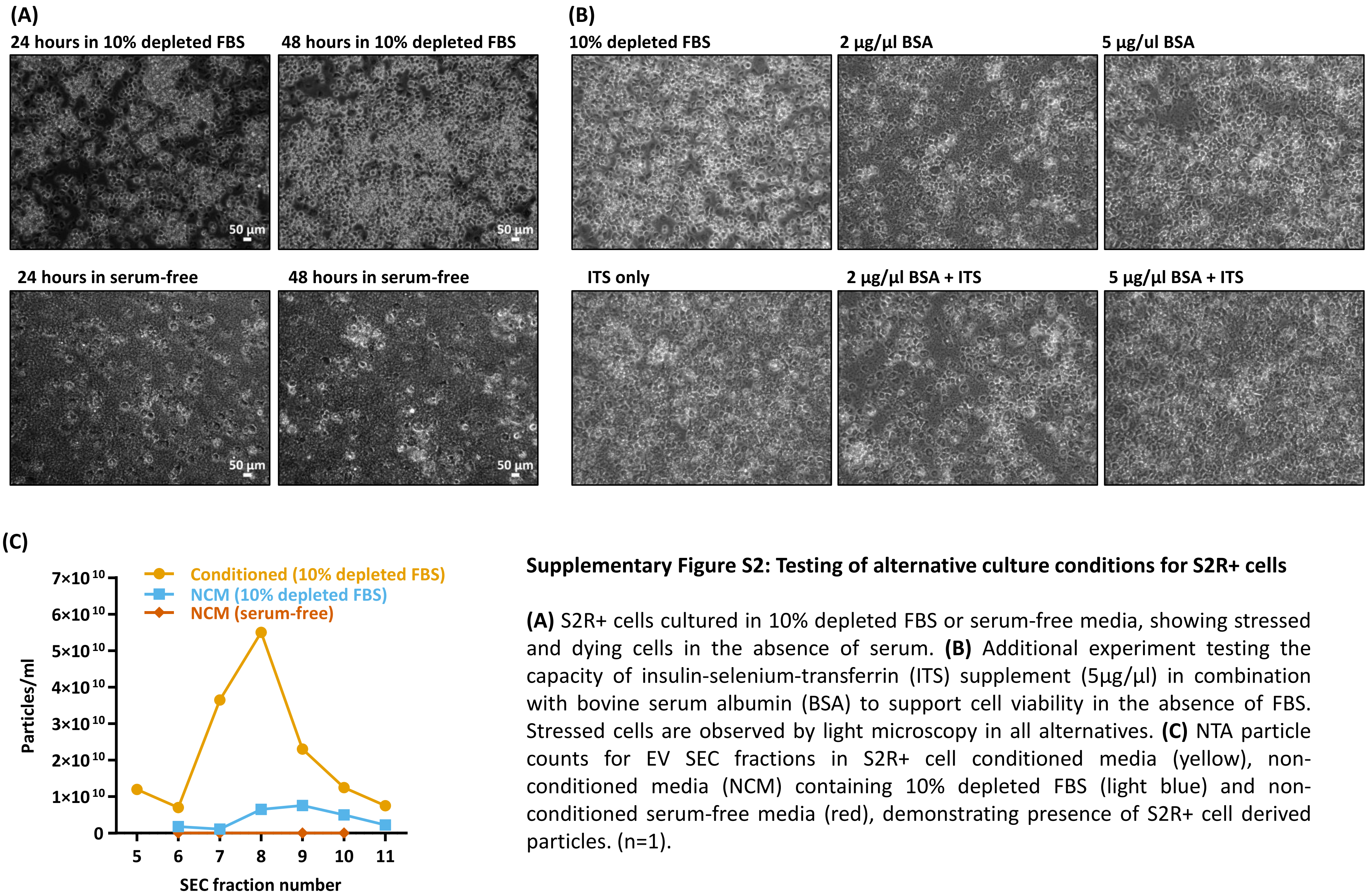

Supplementary Figure S2: Testing of alternative culture conditions for S2R+ cells

(A) S2R+ cells cultured in 10% depleted FBS or serum-free media, showing stressed and dying cells in the absence of serum. **(B)** Additional experiment testing the capacity of insulin-selenium-transferrin (ITS) supplement (5µg/µl) in combination with bovine serum albumin (BSA) to support cell viability in the absence of FBS. Stressed cells are observed by light microscopy in all alternatives. **(C)** NTA particle counts for EV SEC fractions in S2R+ cell conditioned media (yellow), non-conditioned media (NCM) containing 10% depleted FBS (light blue) and non-conditioned serum-free media (red), demonstrating presence of S2R+ cell derived particles. (n=1).

(A)

Reference sequence	Reads mapped to reference																	
	EV control R1	EV Control R2	EV Control R3	Cell Control R1	Cell Control R2	Cell Control R3	EV 2mM R1	EV 2mM R2	EV 2mM R3	Cell 2mM R1	Cell 2mM R2	Cell 2mM R3	EV 0.5mM R1	EV 0.5mM R2	EV 0.5mM R3	Cell 0.5mM R1	Cell 0.5mM R2	Cell 0.5mM R3
Total	25135922	16769682	20105170	24126694	22368262	21672928	20718340	18443406	18017858	21280766	18584410	23599080	20032144	25142152	22120348	17839120	22444988	23536440
rRNA (Bowtie2 end-to-end)	21089788	15691226	16292528	1662020	2783380	912566	18593328	17197048	14402284	3241810	5488346	3664012	16853518	23375806	17554230	1743232	4590348	1280978
Transposon (Bowtie2 end-to-end)	325578	122012	176484	2394170	2368772	1486762	205494	173308	356436	2182392	2145520	2242228	314666	253748	330920	2422368	2535550	1966966
Repeat mask (Bowtie2 end-to-end)	8996	11470	10184	89650	150004	204004	13208	12802	8592	96498	107084	83680	11614	15162	11032	88898	118298	114100
Drosophila genome (HISAT2)	694214	620974	755114	13596078	13717090	9217038	742980	659814	631470	7207420	8675242	8256302	653270	893344	732722	8093208	10890880	9227952
Bovine genome (Bowtie2 end-to-end)	10738	11374	2204	400280	352940	200248	2282	5684	2838	1780412	322280	222298	9512	17838	2870	327536	1590104	163472
Totivirus (Bowtie2 end-to-end)	107140	14026	100720	1102076	384746	3052484	24086	9124	95228	2493718	188794	2616550	86354	22458	86034	1569008	343246	3936234
Alphanodavirus (Bowtie2 end-to-end)	1721256	29366	1742470	1842736	413048	3133014	599738	26048	1461544	1391332	168596	2811508	1313090	46452	2067542	1219820	350998	3199686
Alphanodavirus (Bowtie2 local)	454240	13294	552500	1017490	172482	1705506	175486	40528	558312	974250	125836	1518548	384736	25338	776974	730260	187388	1792580
rRNA (Bowtie2 local)	285804	141718	171380	19080	31080	13954	209438	229342	218028	61434	57958	39058	149076	284580	204736	20640	68140	15756
Drosophila genome (Bowtie2 local)	40838	21736	31484	935556	912220	667724	26878	24850	39390	554976	569928	633708	29004	36816	36594	595990	705312	653354
Totivirus (Bowtie2 local)	4742	652	6362	56478	19088	137442	858	436	4064	95248	9948	103130	2726	1082	4762	65802	16170	156964

(B)

Reference sequence	Percentage of total reads mapped to reference																	
	EV control R1	EV Control R2	EV Control R3	Cell Control R1	Cell Control R2	Cell Control R3	EV 2mM R1	EV 2mM R2	EV 2mM R3	Cell 2mM R1	Cell 2mM R2	Cell 2mM R3	EV 0.5mM R1	EV 0.5mM R2	EV 0.5mM R3	Cell 0.5mM R1	Cell 0.5mM R2	Cell 0.5mM R3
rRNA (Bowtie2 end-to-end)	83.9	93.6	81.0	6.9	12.4	4.2	89.7	93.2	79.9	15.2	29.5	15.5	84.1	93.0	79.4	9.8	20.5	5.4
Transposon (Bowtie2 end-to-end)	1.3	0.7	0.9	9.9	10.6	6.9	1.0	0.9	2.0	10.3	11.5	9.5	1.6	1.0	1.5	13.6	11.3	8.4
Repeat mask (Bowtie2 end-to-end)	0.0	0.1	0.1	0.4	0.7	0.9	0.1	0.1	0.0	0.5	0.6	0.4	0.1	0.1	0.0	0.5	0.5	0.5
Drosophila genome (HISAT2)	2.8	3.7	3.8	56.4	61.3	42.5	3.6	3.6	3.5	33.9	46.7	35.0	3.3	3.6	3.3	45.4	48.5	39.2
Bovine genome (Bowtie2 end-to-end)	0.0	0.1	0.0	1.7	1.6	0.9	0.0	0.0	0.0	8.4	1.7	0.9	0.0	0.1	0.0	1.8	7.1	0.7
Totivirus (Bowtie2 end-to-end)	0.4	0.1	0.5	4.6	1.7	14.1	0.1	0.0	0.5	11.7	1.0	11.1	0.4	0.1	0.4	8.8	1.5	16.7
Alphanodavirus (Bowtie2 end-to-end)	6.8	0.2	8.7	7.6	1.8	14.5	2.9	0.1	8.1	6.5	0.9	11.9	6.6	0.2	9.3	6.8	1.6	13.6
Alphanodavirus (Bowtie2 local)	1.8	0.1	2.7	4.2	0.8	7.9	0.8	0.2	3.1	4.6	0.7	6.4	1.9	0.1	3.5	4.1	0.8	7.6
rRNA (Bowtie2 local)	1.1	0.8	0.9	0.1	0.1	0.1	1.0	1.2	1.2	0.3	0.3	0.2	0.7	1.1	0.9	0.1	0.3	0.1
Drosophila genome (Bowtie2 local)	0.2	0.1	0.2	3.9	4.1	3.1	0.1	0.1	0.2	2.6	3.1	2.7	0.1	0.1	0.2	3.3	3.1	2.8
Totivirus (Bowtie2 local)	0.0	0.0	0.0	0.2	0.1	0.6	0.0	0.0	0.0	0.4	0.1	0.4	0.0	0.0	0.0	0.4	0.1	0.7

Supplementary Figure S3: Alignment statistics for all sequenced samples

(A) Paired reads aligned to each reference sequence, with alignment tool and setting shown in brackets. (B) Percentage of total sequenced reads aligned to each reference.

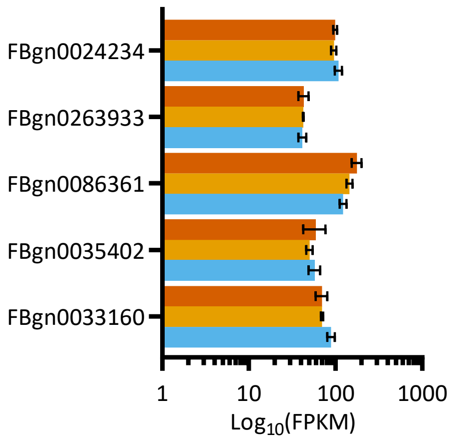

Supplementary Figure S4: mRNAs depleted in high stress EVs versus parental cells in MAPK signalling gene ontology category
FPKM abundance values for 5 mRNAs contributing towards overrepresentation of MAPK signalling terms in gene ontology analysis. No significant differences for these mRNAs were seen by differential expression analysis with DESEQ2 (FDR < 0.05).

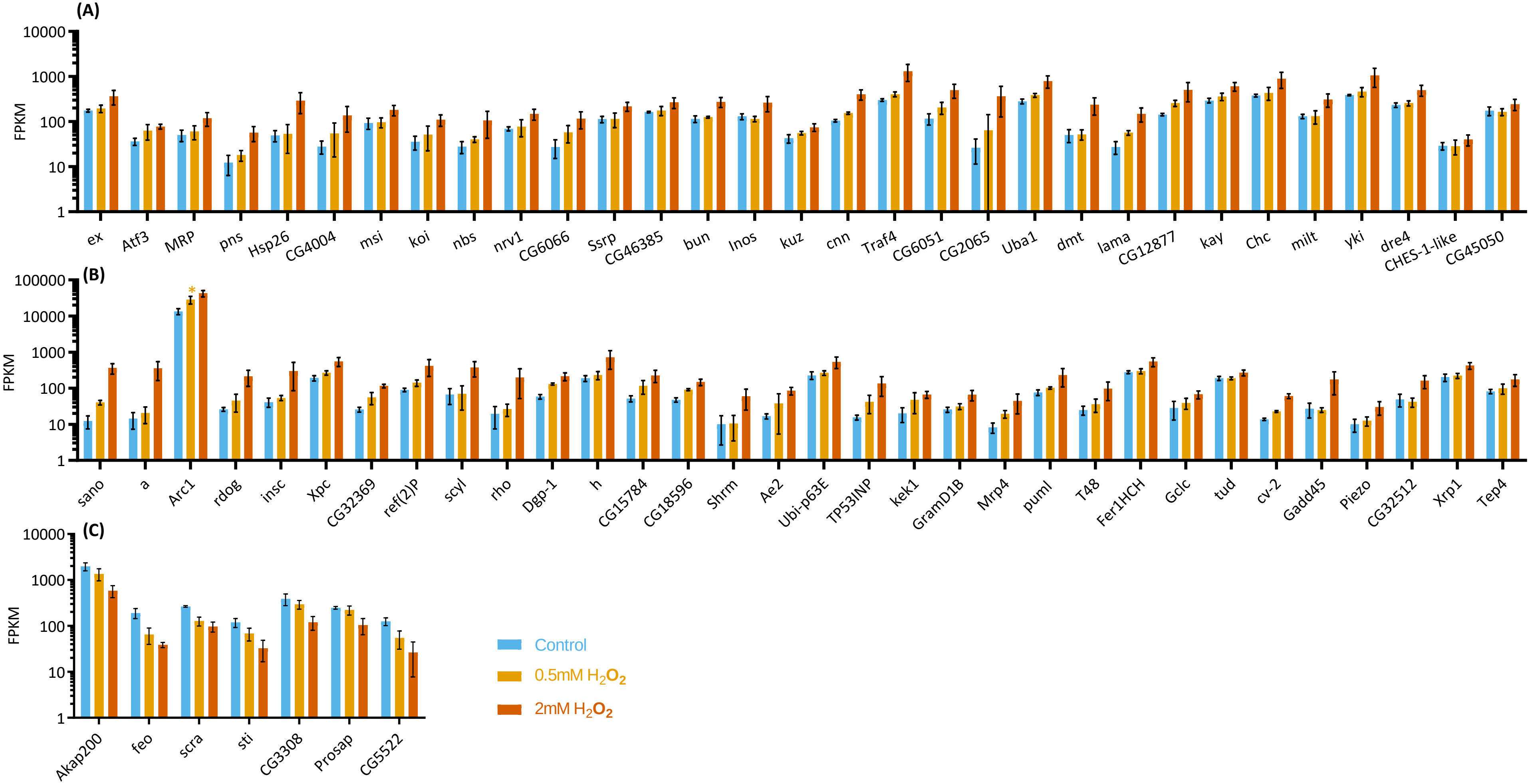

Supplementary Figure S5: Differentially abundant RNAs in stress EVs

(A) FPKM abundance values for 31 mRNAs detected as significantly upregulated in stress EVs versus control EVs, but not stress cells versus control cells (2mM H_2O_2 stress) **(B)** 32 mRNAs upregulated in both EV and cell comparisons. (**C**) downregulated mRNAs in stress EVs, none of which show significant differences in cell comparisons. Differential expression determined via DESEQ2 (FDR < 0.05).

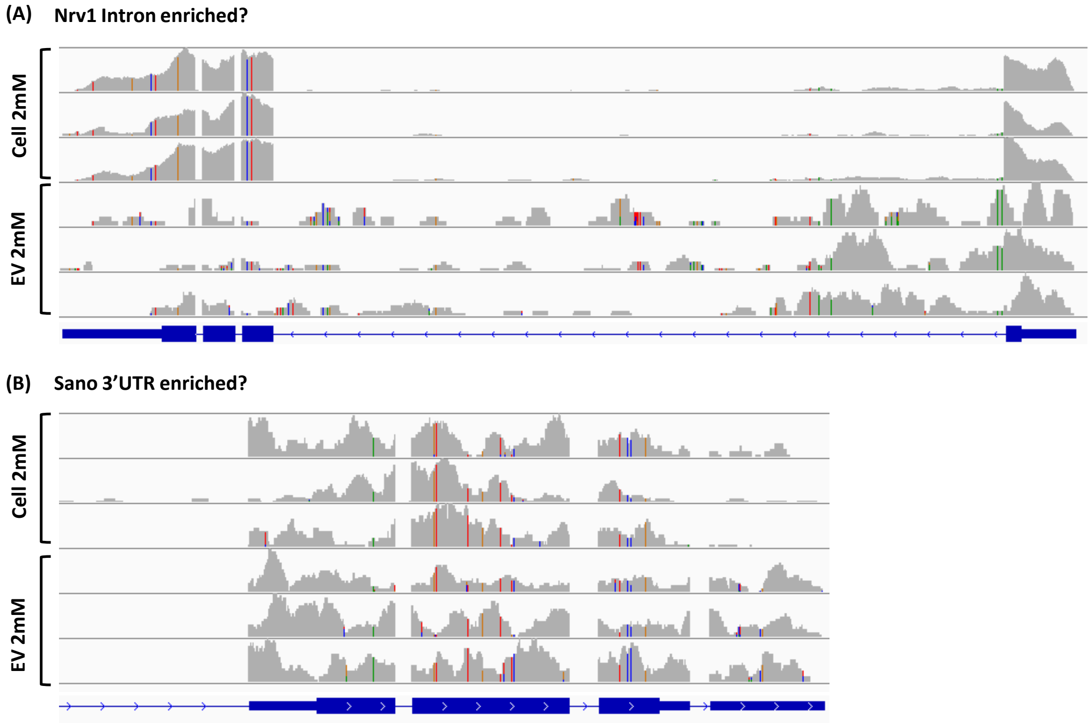

Supplementary Figure S6: Visual representation of regional enrichment in specific mRNAs

(A) and **(B)** Screenshots of read distribution along Nrv1 and Sano loci on Integrative Genome Viewer, showing intron and 3'UTR enrichment respectively, in EV and cell RNA under 2mM H_2O_2 stress.
